# Association between anti-TNF and NSAID therapy and choroidal and macular thickness in ankylosing spondylitis: an OCT-based study

**DOI:** 10.1186/s12886-026-04619-w

**Published:** 2026-01-12

**Authors:** Zarife Ekici Gök, Mehmet Şakir Altuner, Kayhan Mutlu

**Affiliations:** 1 Department of Ophthalmology, Faculty of Medicine, Malatya Turgut Özal University, Malatya, Türkiye; 2 Department of Ophthalmology, Bursa Çekirge State Hospital, Bursa, Türkiye; 3 Department of Ophthalmology, Faculty of Medicine, Aksaray University, Aksaray, Türkiye

**Keywords:** Ankylosing spondylitis, Optical coherence tomography, Choroid, Macular thickness, Anti-TNF therapy

## Abstract

This prospective, cross-sectional study aimed to evaluate the association between non-steroidal anti-inflammatory drugs (NSAIDs) and anti–tumor necrosis factor-α (anti-TNF-α) therapy and retinal and choroidal thickness in patients with ankylosing spondylitis (AS) without uveitis, and to compare these findings with healthy controls. A total of 65 AS patients and 30 age- and sex-matched healthy controls were included. Macular thickness, retinal nerve fiber layer (RNFL) thickness, and subfoveal choroidal thickness were measured using spectral-domain optical coherence tomography (SD-OCT). Patients receiving NSAIDs and/or sulfasalazine (Group 1, *n* = 30) were compared with patients receiving anti-TNF-α therapy (Group 2, *n* = 35) and healthy controls (Group 3, *n* = 30). Mean subfoveal choroidal thickness was significantly greater in Group 1 compared with controls (*p* = 0.026). Nasal inner (*p* = 0.008) and nasal outer (*p* = 0.003) macular subfield thicknesses were significantly lower in both AS groups compared with controls, while RNFL thickness did not differ significantly among groups (*p* = 0.069). Analysis of covariance (ANCOVA) showed that age and disease duration had no significant effect on these outcomes. These findings indicate that choroidal and macular thickness measurements are associated with AS even in the absence of clinically evident uveitis, and that anti-TNF-α therapy is associated with lower choroidal thickness compared with NSAID treatment. OCT-based choroidal assessment may provide additional information on ocular structural changes associated with AS; however, its role in treatment monitoring requires confirmation in longitudinal studies.

## Introduction

Ankylosing spondylitis (AS) is a chronic inflammatory rheumatic disease that primarily affects the spine, peripheral joints, and entheses [1]. Uveitis represents the most frequent extra-articular manifestation, and the reported prevalence of anterior uveitis in AS ranges between 30% and 40% [2]. Although ocular involvement in spondyloarthropathies is predominantly characterized by anterior uveitis, the choroid—one of the most vascular layers of the uveal tract—may also undergo changes in the context of systemic inflammation. Spectral-domain optical coherence tomography (SD-OCT) provides a noninvasive and repeatable method for evaluating choroidal structure and thickness [3]. In clinical practice, OCT is widely used to assess posterior segment parameters, including macular thickness, retinal volume, retinal nerve fiber layer (RNFL) thickness, and choroidal thickness [4].

Cytokines including tumor necrosis factor-α (TNF-α), interleukin (IL)-1, IL-6, IL-17, and IL-23 play key roles in the pathogenesis of AS. Among these, TNF-α has been strongly implicated in inflammatory, exudative, neovascular, and neurodegenerative ocular responses [5]. Anti–tumor necrosis factor-α (anti-TNF-α) agents have demonstrated substantial efficacy in controlling both systemic and ocular inflammation [6–8]. Current treatment strategies aim to relieve pain and suppress inflammation, and TNF-α inhibitors such as infliximab, etanercept, adalimumab, and golimumab are commonly used in patients with an inadequate response to NSAIDs or conventional disease-modifying agents, including sulfasalazine [9].

The aim of this study was to evaluate posterior segment alterations in AS patients without uveitis and to compare retinal and choroidal thickness parameters among patients receiving different anti-inflammatory treatments and healthy control**s.**

## Materials and methods

This prospective, cross-sectional study included 95 participants: 65 patients with AS and 30 healthy controls. AS patients were recruited from the rheumatology clinic during routine follow-up and referred for ophthalmologic evaluation. Group 1 consisted of 30 patients treated with NSAIDs and/or sulfasalazine, Group 2 included 35 patients receiving anti-TNF-α therapy, and Group 3 consisted of 30 age- and sex-matched healthy individuals.

All AS diagnoses were made by a rheumatologist according to the modified New York criteria [10]. Only patients in clinical remission were included, defined as the absence of active uveitis or ocular symptoms and an Ankylosing Spondylitis Disease Activity Score (ASDAS) < 2.1, corresponding to low disease activity. None had active uveitis at evaluation; one patient in the anti-TNF group had a prior history of uveitis. Systemic treatment had to be stable for at least three months prior to imaging.

Written informed consent was obtained from all participants, and the study adhered to the principles of the Declaration of Helsinki. Ethical approval was obtained from the Malatya Turgut Özal University Clinical Research Ethics Committee (Approval No: 2022/55).

A comprehensive ophthalmologic examination was performed by the same ophthalmologist and included best-corrected visual acuity assessment using Snellen charts, slit-lamp biomicroscopy, intraocular pressure measurement, and dilated fundus examination using a 90-D non-contact lens. Spectral-domain optical coherence tomography (SD-OCT) was used to measure macular thickness, peripapillary retinal nerve fiber layer (RNFL) thickness, and subfoveal choroidal thickness. All OCT measurements were obtained between 09:00 and 12:00 to minimize the effects of diurnal variation. To avoid bias related to inter-eye correlation, only one eye per participant was included in the analysis [11].

### Inclusion criteria

- Age between 18 and 50 years.
- Best-corrected visual acuity ≥ 20/20.
- Spherical equivalent < ± 3.0 diopters.
- Axial length < 25 mm.
- No history of ocular surgery.
- No ocular or neurological disease.
- Clinical remission at the time of imaging.
- Stable systemic treatment for ≥ 3 months.
- Minimum of six months of NSAID, sulfasalazine, or anti-TNF-α therapy.

### Exclusion criteria

- Spherical equivalent ≥ ± 3.0 diopters.
- Glaucoma, ocular hypertension, or optic neuropathy.
- Retinopathy or other retinal disease.
- History of ocular trauma or intraocular surgery.
- Systemic corticosteroid use.
- Presence of other systemic inflammatory or vascular diseases.

At the time of initial diagnosis, acute-phase reactant levels may vary among patients with AS [5]. However, as all ophthalmologic and OCT measurements were performed during the remission period and due to the heterogeneity of acute-phase reactant values at diagnosis, inflammatory markers such as C-reactive protein (CRP) and erythrocyte sedimentation rate (ESR) were not included in the comparative analyses.

Demographic characteristics, disease duration, treatment modality, and history of uveitis were recorded for all participants. OCT findings were evaluated in relation to these variables.

Patients in Group 2 were receiving anti–tumor necrosis factor-α (anti-TNF-α) therapy at the time of ophthalmologic evaluation. The anti-TNF agents included adalimumab, infliximab, golimumab, certolizumab pegol, and etanercept. All patients had been receiving anti-TNF-α therapy for at least six months prior to OCT imaging. Treatments were administered according to standard rheumatologic dosing regimens. Due to the prospective cross-sectional design of the study, minor variations in treatment duration and dosing schedules were present among patients; however, treatment stability prior to imaging was ensured.

### Macular, RNFL, and subfoveal choroidal thickness measurements

All measurements were obtained using a spectral-domain OCT device (RS-3000 Advance, NIDEK, Japan). For each participant, the scan with the highest signal strength (≥ 7) was selected for analysis. Peripapillary RNFL thickness was measured using a 6 × 6 mm² scan centered on the optic nerve head, and the global average RNFL value was used for statistical analysis.

**Fig. 1:**
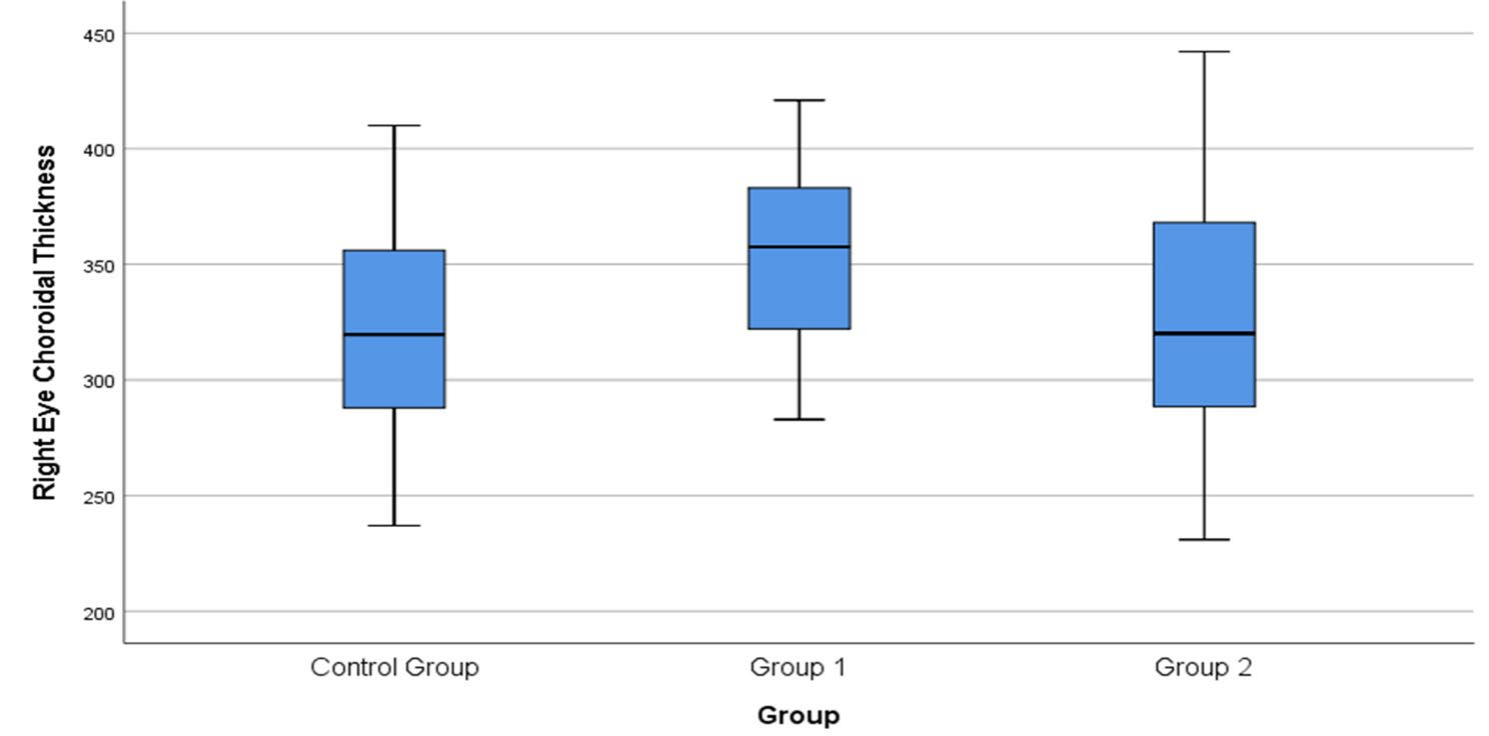
Box plot showing the comparison of subfoveal choroidal thickness in the eyes among the three study groups (Control, Group 1: NSAIDs, Group 2: Anti-TNF-α). The central line represents the median; the box, the interquartile range; and the whiskers, the minimum and maximum values

**Fig. 2:**
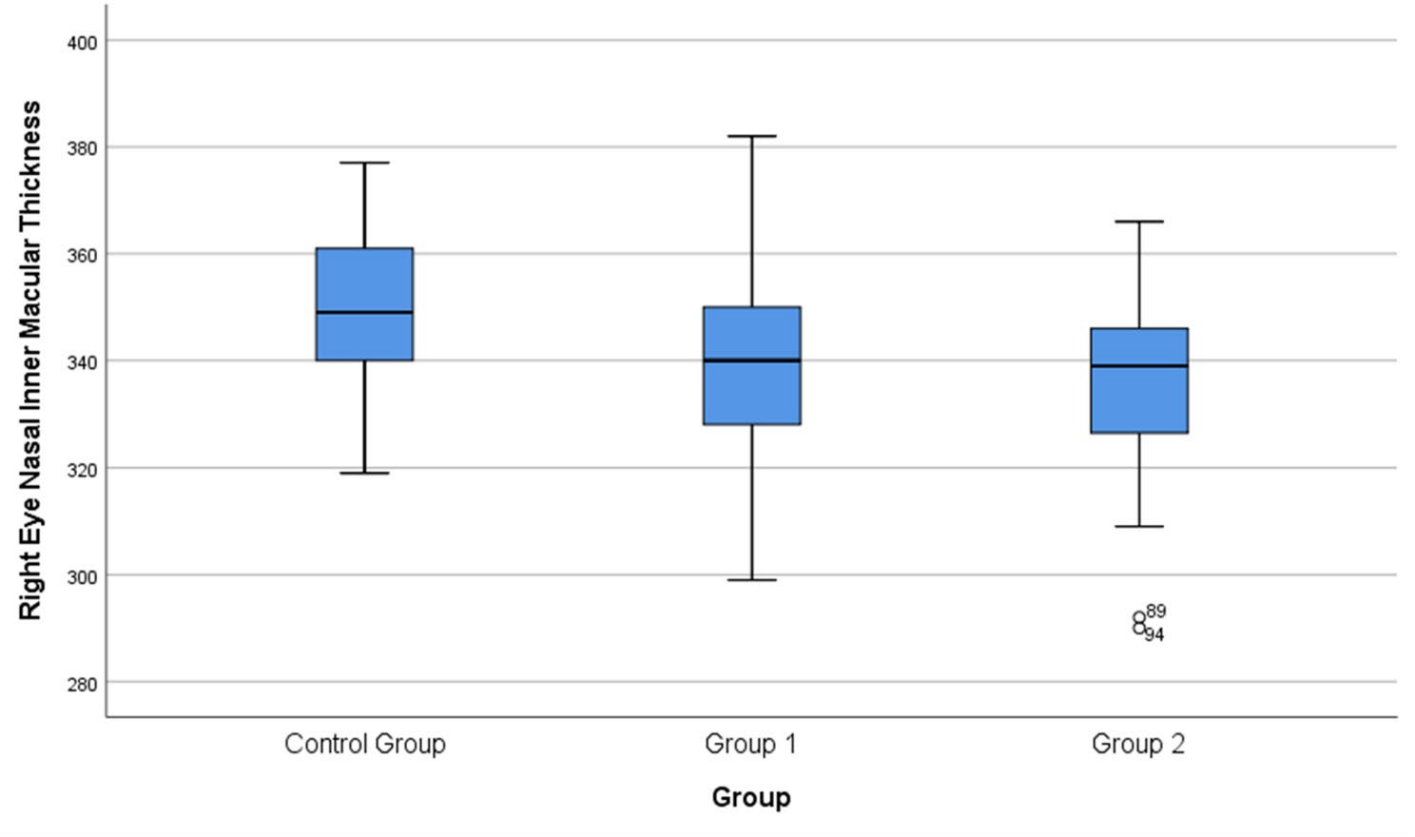
Box plot showing the comparison of nasal inner macular thickness in the eyes among the three study groups (Control, Group 1: NSAIDs, Group 2: Anti-TNF-α). The central line represents the median; the box, the interquartile range; and the whiskers, the minimum and maximum values. Outliers are shown as separate points

**Fig. 3:**
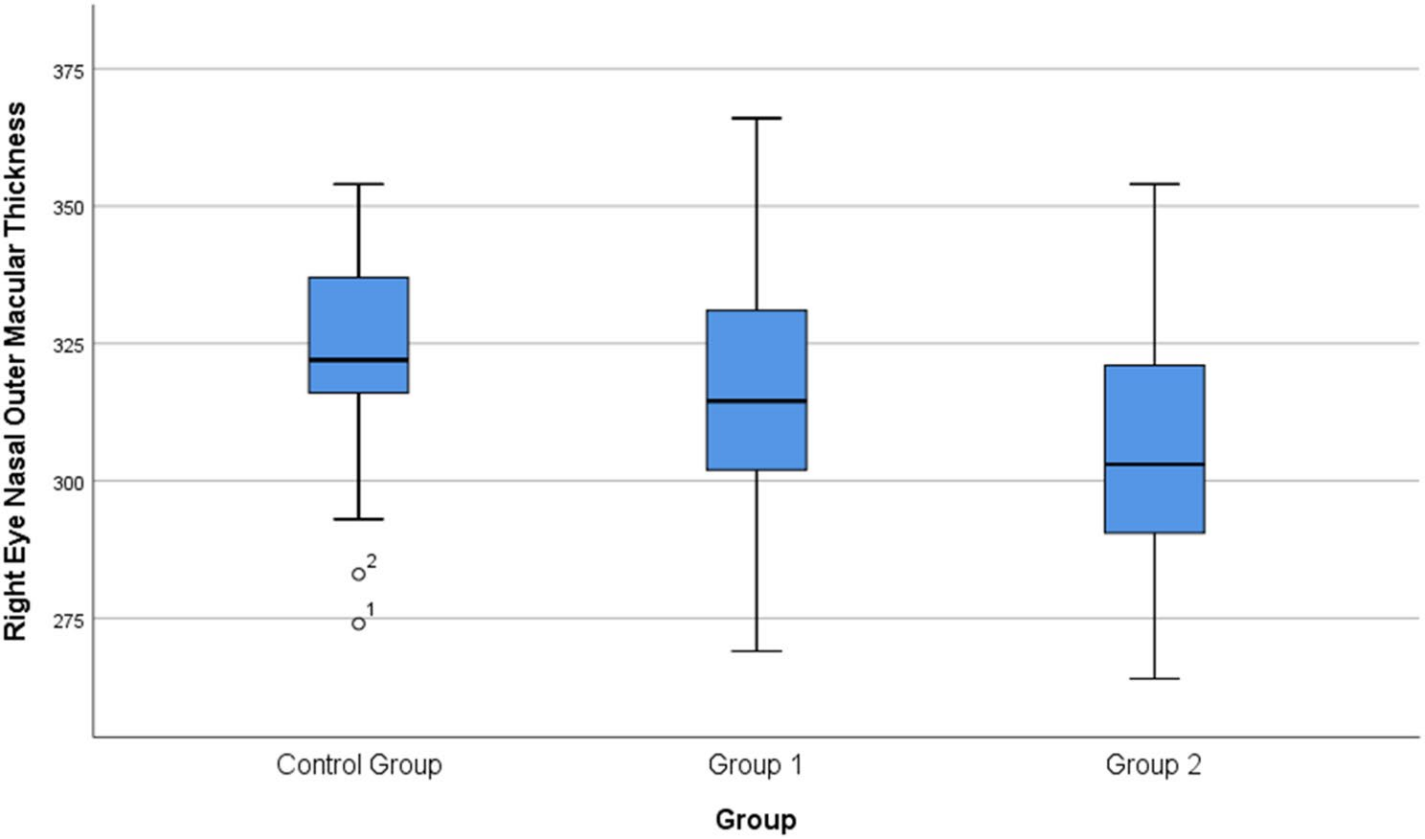
Box plot showing the comparison of nasal outer macular thickness in the eyes among the three study groups (Control, Group 1: NSAIDs, Group 2: Anti-TNF-α). The central line represents the median; the box, the interquartile range; and the whiskers, the minimum and maximum values. Outliers are displayed as separate points

Macular thickness measurements were obtained in nine regions defined by the Early Treatment Diabetic Retinopathy Study (ETDRS) grid using the device software. Subfoveal choroidal thickness was measured using enhanced depth imaging OCT (EDI-OCT) and defined as the vertical distance from the hyperreflective line of Bruch’s membrane to the hyperreflective line corresponding to the inner surface of the sclera. All images were acquired by the same experienced technician (Figs. 1, 2, and 3).

### Statistical analysis

Statistical analyses were primarily performed using SPSS software (IBM SPSS Statistics, v23). Jamovi software (The Jamovi Project, v2.4) was additionally used for data visualization and confirmation of selected statistical results. The distribution of variables was assessed with the Shapiro–Wilk test in order to determine the appropriate statistical tests for intergroup comparisons. Parametric tests were applied to variables that met the normality assumption, while non-parametric tests were applied to those that did not. The level of statistical significance was accepted as 0.05.

For variables with normal distribution, ANOVA was used, whereas for those without normal distribution, the Kruskal–Wallis test was applied. Following ANOVA, post hoc group comparisons were performed with the Bonferroni test, while for the Kruskal–Wallis test, pairwise comparisons were conducted with the Bonferroni-corrected Mann–Whitney U test.

Post hoc pairwise comparisons were performed only for variables that showed statistically significant differences in the overall three-group analysis.

### Power analysis

An a priori power analysis using G*Power (effect size d = 0.6, α = 0.05) demonstrated that a minimum of 27 participants per group was required to achieve 80% power. The study exceeded this requirement for all groups.

A p-value < 0.05 was considered statistically significant.

## Results

There were no statistically significant differences among the groups in terms of age, sex distribution, smoking status, intraocular pressure, or spherical equivalent values (*p* > 0.05 for all). The mean ages were 38.03 years in Group 1, 40.2 years in Group 2, and 31 years in Group 3. Demographic characteristics are summarized in Table 1. All AS patients met the ASDAS-based criteria for low disease activity at the time of ophthalmologic evaluation.

**Table 1:** Demographic and clinical characteristics of the study groups

Variable	Group 1 (NSAIDs/SS)	Group 2 (Anti-TNF-α)	Group 3 (Controls)
Gender, n (%)	Female: 17 (56.7%) Male: 13 (43.3%)	Female: 13 (37.1%) Male: 22 (62.9%)	Female: 15 (50%) Male: 15 (50%)
Age (years), Mean ± SD	38.03 ± 9.84 (18–57)	40.20 ± 11.16 (20–59)	31.00 ± 8.01 (20–49)
AS duration (years), Mean ± SD	5.40 ± 5.44	8.16 ± 6.39	—

Abbreviations: SD = Standard deviation; AS = Ankylosing spondylitis; SS = Sulfasalazine

Only one patient in the anti-TNF-α group had a history of uveitis. In Group 1, 9 patients used sulfasalazine in combination with NSAIDs, whereas 21 used NSAIDs alone. In Group 2, the distribution of anti-TNF-α agents was as follows: certolizumab pegol (*n* = 4), golimumab (*n* = 9), adalimumab (*n* = 20), etanercept (*n* = 1), and infliximab (*n* = 1).

The mean subfoveal choroidal thickness values were 353.23 ± 38.14 μm in Group 1, 328.80 ± 53.23 μm in Group 2, and 322.63 ± 43.35 μm in Group 3. Overall intergroup comparison revealed a statistically significant difference among the groups (*p* = 0.026) (Table 2). Bonferroni-adjusted post hoc analyses demonstrated that subfoveal choroidal thickness was significantly greater in Group 1 compared with Group 3 (Table 3).

**Table 2:** Comparison of macular thickness, RNFL thickness and subfoveal choroidal thickness among the three groups

ETDRS Areas (µm)	Group 1 Mean ± SD (µm)	Group 2 Mean ± SD (µm)	Group 3 Mean ± SD (µm)	*p*-value
Superior-inner	342.17 ± 19.55	341.34 ± 17.67	345.70 ± 23.49	0.396
Temporal-inner	331.30 ± 14.38	334.11 ± 21.63	336.50 ± 13.99	0.510
Inferior-inner	338.87 ± 17.85	336.74 ± 22.24	346.37 ± 14.30	0.105
Nasal-inner	340.63 ± 17.46	335.97 ± 17.38	349.87 ± 14.56	0.008
Superior-outer	309.17 ± 14.99	304.91 ± 14.40	310.57 ± 14.43	0.402
Temporal-outer	298.27 ± 18.63	304.40 ± 20.32	297.53 ± 12.97	0.230
Inferior-outer	297.60 ± 22.40	298.31 ± 21.44	300.97 ± 17.08	0.479
Nasal-outer	315.30 ± 23.20	305.63 ± 21.12	323.60 ± 18.73	0.003
Central macula	259.47 ± 23.60	258.43 ± 26.66	267.53 ± 19.91	0.356
RNFL (µm)	108.23 ± 12.13	111.40 ± 10.11	114.77 ± 10.14	0.069
Subfoveal choroid	353.23 ± 38.14	328.80 ± 53.23	322.63 ± 43.35	0.026

Abbreviations: RNFL = Retinal nerve fiber layer; SD = Standard deviation; ETDRS = Early Treatment Diabetic Retinopathy Study

Data are presented as mean ± standard deviation. Overall intergroup comparisons were performed using one-way analysis of variance (ANOVA) for normally distributed variables and the Kruskal–Wallis test for non-normally distributed variables. Only variables with statistically significant overall differences were subjected to Bonferroni-adjusted post hoc pairwise comparisons, which are presented in Table 2. RNFL = retinal nerve fiber layer; SD = standard deviation; ETDRS = Early Treatment Diabetic Retinopathy Study

**Table 3:** Pairwise comparisons for parameters showing significant overall intergroup differences

Parameter	Group comparison	Mean ± SD (Group A)	Mean ± SD (Group B)	Adjusted *p*-value
Nasal-inner macular thickness (µm)	Group 1 vs. Group 3	340.63 ± 17.46	349.87 ± 14.56	0.031
Nasal-inner macular thickness (µm)	Group 2 vs. Group 3	335.97 ± 17.38	349.87 ± 14.56	0.002
Nasal-outer macular thickness (µm)	Group 1 vs. Group 3	315.30 ± 23.20	323.60 ± 18.73	0.045
Nasal-outer macular thickness (µm)	Group 2 vs. Group 3	305.63 ± 21.12	323.60 ± 18.73	0.001
Subfoveal choroidal thickness (µm)	Group 1 vs. Group 3	353.23 ± 38.14	322.63 ± 43.35	0.034

Only parameters showing statistically significant differences in the overall three-group analysis were included. Pairwise comparisons were performed using Bonferroni-adjusted post hoc tests. Group 1: NSAIDs and/or sulfasalazine; Group 2: anti-TNF-α therapy; Group 3: healthy controls

RNFL thickness did not differ significantly among the groups (*p* = 0.069), with mean values of 108.23 ± 12.13 μm in Group 1, 111.40 ± 10.11 μm in Group 2, and 114.77 ± 10.14 μm in Group 3 (Table 2).

Regarding macular thickness, significant intergroup differences were observed in the nasal inner (*p* = 0.008) and nasal outer (*p* = 0.003) ETDRS subfields (Table 2). Bonferroni-adjusted post hoc analyses showed that both AS groups had significantly lower nasal inner and nasal outer macular thickness compared with healthy controls (Table 3).

Analysis of covariance (ANCOVA) was performed to assess whether the variables showing significant overall differences were independent of potential confounders such as age and disease duration. Subfoveal choroidal thickness and nasal outer macular thickness remained significantly different among the groups after adjustment, whereas age and disease duration showed no significant effects (*p* > 0.05 for all).

Overall, these findings indicate that subfoveal choroidal thickness and nasal macular thickness are associated with ankylosing spondylitis and differ according to anti-inflammatory treatment modality, independent of age and disease duration.

## Discussion

The uveal tissue, as the vascular layer of the eye, is susceptible to systemic inflammatory processes in ankylosing spondylitis (AS), most commonly presenting clinically as acute anterior uveitis [12]. Although anterior uveitis represents the hallmark ocular manifestation of AS, several studies have demonstrated that posterior segment structures may also exhibit structural alterations, even when anterior segment findings are unremarkable. In some cases, macular edema or subtle posterior segment changes have been detected by OCT despite a quiet anterior chamber, suggesting posterior involvement beyond clinically evident anterior uveitis [13, 14].

The choroid, which contains fenestrated vessels, stromal melanocytes, fibroblasts, and a dense network of resident immune cells, plays an essential role in ocular homeostasis. These immune cells contribute to the regulation of inflammatory responses within the retina and choroid [15]. In addition, the choroidal vasculature demonstrates autoregulatory capacity in response to systemic blood pressure, intraocular pressure, and autonomic influences, rendering it sensitive to systemic disease states [16]. Consistent with this, previous studies have reported alterations in choroidal thickness across a range of ocular and systemic conditions, including inflammatory diseases, with measurable changes observed during active inflammatory episodes such as acute anterior uveitis [17–19].

In AS patients without uveitis, increased subfoveal choroidal thickness compared with healthy individuals has been previously demonstrated [20]. In our study, choroidal thickness was significantly higher in patients treated with NSAIDs and sulfasalazine compared with those receiving anti-TNF-α therapy, supporting the hypothesis that inflammatory activity may be associated with increased choroidal thickness in AS. If increased choroidal thickness is considered an indicator of subclinical inflammation, the lower values observed in patients receiving anti-TNF-α therapy suggest an association between treatment modality and choroidal thickness. Although none of the patients exhibited active uveitis at the time of imaging, these structural differences support the concept that the choroid may reflect subclinical inflammatory activity, even during clinical remission.

However, in the absence of concurrent laboratory inflammatory markers or vascular imaging parameters, these findings should be interpreted as structural correlates that may be associated with subclinical inflammatory processes rather than direct evidence of ongoing inflammation.

Previous studies support the notion that choroidal thickness may change in response to disease activity and treatment. In a study comparing uveitic eyes, fellow non-uveitic eyes, and healthy controls during active and remission phases, subfoveal choroidal thickness was significantly increased during active disease but did not differ during remission [21]. A prospective study evaluating the effects of nine months of anti-TNF-α therapy on retinal and macular parameters in AS patients reported no significant structural changes [22]. Another study involving AS patients with acute anterior uveitis but no clinically apparent posterior involvement demonstrated retinal thinning, suggesting subtle posterior segment alterations possibly related to previous inflammatory episodes [23].

Steiner et al. recently demonstrated that increased choroidal thickness may serve as a biomarker associated with treatment response in patients with ankylosing spondylitis. In their study, choroidal thickness decreased following effective anti-inflammatory therapy, supporting the concept of subclinical ocular involvement even in the absence of clinically evident uveitis [24]. Our findings are consistent with these observations and further support the concept that OCT-derived choroidal parameters may reflect posterior segment structural changes associated with different treatment strategies in AS.

Karataş et al. evaluated retinal thickness and microvascular changes using OCTA in patients with spondyloarthritides receiving NSAIDs and anti–TNF-α therapy. Their findings demonstrated treatment-related differences in retinal parameters and vascular density, supporting the presence of subclinical retinal involvement in inflammatory rheumatic diseases [25]. Although OCTA was not performed in the present study, our results regarding choroidal and macular thickness changes are consistent with the concept of subclinical ocular involvement described in OCTA-based studies.

Although posterior segment involvement in ankylosing spondylitis has been previously investigated, the present study provides additional insight by focusing exclusively on patients in clinical remission defined by ASDAS criteria and without active uveitis. In contrast to earlier reports that evaluated AS patients as a single, heterogeneous group, our study compares different anti-inflammatory treatment modalities within a remission cohort. This design allows a more refined assessment of treatment-associated differences in posterior segment structural parameters using OCT-derived choroidal and macular measurements.

In our study, statistically significant thinning of the nasal macular subfields was observed in AS patients compared with healthy controls. Given that the mean ASDAS score was low and only one patient had a documented history of uveitis, no clear clinical correlate could be identified for this finding. Therefore, nasal macular thinning in this cohort may represent a subtle structural variation associated with posterior segment involvement in AS rather than overt inflammatory damage.

The nasal macula is anatomically adjacent to the optic nerve head and contains a higher density of retinal nerve fiber layers, rendering this region potentially more vulnerable to microvascular, hemodynamic, or inflammatory influences [26, 27]. Previous studies evaluating choroidal and retinal perfusion have also demonstrated distinct autoregulatory properties in the nasal macular region, suggesting that it may respond differently to systemic inflammatory or autonomic stimuli [28]. Accordingly, the selective thinning observed in the nasal ETDRS subfields in our study may reflect region-specific susceptibility of the posterior segment in AS patients, even during periods of clinical remission. Although these differences were statistically significant, their clinical relevance remains uncertain, as functional assessments such as visual field testing or electrophysiological evaluation were not performed. These findings should therefore be interpreted as subclinical structural variations rather than clinically actionable pathology.

Analysis of covariance confirmed that age and disease duration did not significantly influence choroidal or nasal macular thickness. This finding suggests that the observed differences between treatment groups are independent of these demographic or disease-related factors.

Several studies have investigated retinal layer measurements in AS patients receiving anti-TNF therapy. One study reported no significant differences in ganglion cell or retinal layer thickness before and after treatment [22]. Another study comparing NSAID, sulfasalazine, and anti-TNF-α therapy groups found no significant differences in RNFL thickness [29]. In line with these reports, our study likewise did not demonstrate significant differences in RNFL thickness among the different treatment groups.

### Limitations

This study has several limitations. First, its single-center design may limit the generalizability of the findings, and the relatively small sample size may reduce statistical power for detecting subtle differences between treatment groups. In addition, long-term follow-up data regarding the future development of uveitis were limited, despite all patients being in clinical remission at the time of evaluation.

Second, the anti-TNF group was heterogeneous and included both monoclonal antibodies and etanercept, which are known to differ in ocular efficacy. Due to sample size constraints and the cross-sectional design, subgroup analyses and standardization of treatment duration or dosing were not feasible.

Third, the absence of concurrent laboratory inflammatory parameters at the time of OCT imaging precluded direct correlations between systemic inflammatory activity and ocular structural findings. Finally, although retinal parameters were obtained using automated measurements, choroidal thickness is a relatively labile parameter influenced by multiple physiological and systemic factors. The choroidal vascular index (CVI), which may provide a more refined assessment of choroidal vascular involvement, was not evaluated and should be considered in future prospective studies.

## Conclusion

In conclusion, this study demonstrates an association between choroidal and retinal thickness measurements and ankylosing spondylitis, even in the absence of clinically evident uveitis. Differences in OCT-derived parameters were observed among patients receiving different anti-inflammatory treatment modalities; however, due to the cross-sectional design, these findings should be interpreted as associations rather than treatment effects. OCT-based assessment of the choroid may provide additional information on posterior segment structural changes in ankylosing spondylitis; however, its role in treatment monitoring requires confirmation in prospective longitudinal studies with larger cohorts.

## Data Availability

The datasets generated and/or analyzed during the current study are available from the corresponding author upon reasonable request.

## Abbreviations

AS: Ankylosing spondylitis
OCT: Optical coherence tomography
CMT: Central macular thickness
RNFL: Retinal nerve fiber layer
SFCT: Subfoveal choroidal thickness
NSAID: Non-steroidal anti-inflammatory drug
TNF-α: Tumor necrosis factor-alpha
